# Clinical scenarios of suboptimal response to rituximab in relapsing ANCA-associated vasculitis

**DOI:** 10.3389/fmed.2026.1874867

**Published:** 2026-08-31

**Authors:** Sergio Prieto-González, Ellen Romich, Peter A. Merkel, David R. W. Jayne, Rona M. Smith, RITAZAREM investigators, Giles Walters, Chen Au Peh, Dwarakanathan Ranganathan, Simon Carette, Nader Khalidi, Vladamir Tesar, Michael Clarkson, Augusto Vaglio, Shunsuke Furuta, Tomomi Endo, Yoshihiro Arimura, Yoshinori Komagata, Toshiko Ito-Ihara, Hajime Kono, Kazuo Suzuki, Shunya Uchida, Yoshitomo Hamano, Shouichi Fujimoto, Janak de Zoysa, Annette Bruchfeld, Sarah L awman, Rachel B. Jones, David Jayne, Kim Mynard, Rona M. Smith, Simon Bond, Rainer Klocke, Tim Doulton, Charles Pusey, Brian Camilleri, Jacqueline Andrews, Alastair Ferraro, Peter Lanyon, Raashid Luqmani, Caroline Wroe, Lorraine Harper, Reem Al-jayyousi, Chee Kay Cheung, Lindsy Forbess, Carol A. Langford, Robert Spiera, Ulrich Specks, Ora Gewurz-Singer, Vimal Derebail, Patrick Nachman, Naomi Amudala, Carol A. McAlear, Peter A. Merkel, Rennie L. Rhee, Antoine G. Sreih, Larry W. Moreland, Curry L. Koening

**Affiliations:** Royal Adelaide Hospital, Adelaide, South Australia; Royal Brisbane and Women’s Hospital, Herston, Queensland; Mount Sinai Hospital, University of Toronto, Toronto, Ontario, Canada; Christian Pagnoux, McMaster University, Hamilton, Ontario, Canada; Czech Republic: Charles University, Praha, Czech Republic; Cork University Hospital, Cork, Ireland; University of Parma, Parma, ltaly; Chiba University, Chiba, Japan; Kitano Hospital, Osaka, Japan; Eri Muso, Tatsuo Tsukamoto, Kyorin University School of Medicine, Tokyo, Japan; The Clinical and Translational Research Center, Kyoto Prefectural University of Medicine, Kyoto, Japan; Okayama University, Okayama, Japan: Ken-ei Sada, Teikyo University Hospital, Tokyo, Japan; Tokyo Metropolitan Geriatric Hospital, Tokyo, Japan; University of Miyazaki, Miyazaki, Japan; North Shore Hospital, Auckland, New Zealand; Karolinska University Hospital and Karolinska Institute, Stockholm, Sweden; Brighton Royal Sussex County Hospital, Brighton; Cambridge University Hospitals NHS Foundation Trust, Cambridge, UK; Cambridge Clinical Trials Unit, Cambridge University Hospitals NHS Foundation Trust, Cambridge, UK; Marianna Nodale, Dudley Group of Hospitals NHS Trust, Dudley, UK; East Kent Hospitals University NHS Foundation Trust, Canterbury, UK; Imperial College London, London, UK; Ipswich Hospital NHS Trust, Ipswich, UK; Richard Smith, Richard Watts, Leeds Teaching Hospitals Trust, Leeds, UK; Nottingham University Hospital, Nottingham, UK; Nuffield Department of Orthopedics, Rheumatology and Musculoskeletal Science (NDORMs), University of Oxford, Oxford, UK; South Tees Hospitals NHS Foundation Trust, Middlesbrough, UK; University of Birmingham, Birmingham, UK; University Hospitals of Leicester NHS Trust, Leicester, UK; Cedars-Sinai Medical Center, Los Angeles, California, USA; Cleveland Clinic Foundation, Cleveland, Ohio, USA; Hospital for Special Surgery, New York, New York, USA; Mayo Clinic, Rochester, Minnesota, USA; University of Michigan, Ann Arbor, Michigan, USA; University of North Carolina at Chapel Hill, Chapel Hill, North Carolina, USA; University of Pennsylvania Perelman School of Medicine, Philadelphia, Pennsylvania, USA; University of Pittsburgh, Pittsburgh, Pennsylvania; University of Utah, Salt Lake City, Utah; 1 Department of Medicine, University of Cambridge, Cambridge, United Kingdom; 2 Vasculitis and Lupus Clinic, Addenbrooke’s Hospital, Cambridge University Hospitals NHS Foundation Trust, Cambridge, United Kingdom; 3 Vasculitis Research Group, Department of Autoimmune Diseases, Hospital Clínic of Barcelona, IDIBAPS, Barcelona, Spain; 4 Division of Rheumatology, Department of Medicine, University of Pennsylvania, Philadelphia, PA, United States; 5 Division of Epidemiology, Department of Biostatistics, Epidemiology, and Informatics, University of Pennsylvania, Philadelphia, PA, United States

**Keywords:** ANCA-associated vasculitis, granulomatosis with polyangiitis, induction therapy, maintenance therapy, microscopic polyangiitis, PR3-ANCA, relapse, rituximab

## Abstract

**Background:**

Rituximab is effective for induction and maintenance of remission in relapsing ANCA-associated vasculitis (AAV), but some patients do not achieve complete remission or experience relapse despite treatment. We aimed to describe the frequency, characteristics, and outcomes of patients with suboptimal response to rituximab within the RITAZAREM trial.

**Methods:**

*Post hoc* descriptive analysis, including patients with granulomatosis with polyangiitis (GPA) or microscopic polyangiitis (MPA) treated with rituximab for induction (*N* = 188) and those receiving rituximab maintenance (*N* = 85). Three scenarios of suboptimal response were examined: (i) failure to achieve protocol-defined remission at month 4 (*n* = 6); (ii) incomplete remission at month 4, defined as BVAS/WG = 1 (*n* = 10); and (iii) relapse during rituximab maintenance (*n* = 13). Data were summarized descriptively. Time to relapse from month 4 in patients with BVAS/WG = 1 versus BVAS/WG = 0 was explored using Kaplan–Meier analysis.

**Results:**

Six of 188 patients (3%) did not achieve protocol-defined remission by month 4, 10 (5%) had residual low-grade disease activity (BVAS/WG = 1), and 13 of 85 patients (15%) receiving rituximab maintenance relapsed within 24 months during maintenance treatment. All patients with BVAS/WG = 1 at month 4 had a history of PR3-ANCA positivity and ear/nose/throat (ENT) baseline manifestations. Relapse occurred more frequently in this subgroup than in patients with BVAS/WG = 0, and time-to-relapse analysis showed shorter relapse-free survival, although interpretation is limited by the small sample size. Most first relapses were minor, and some patients subsequently experienced additional relapses, including major relapses.

**Conclusion:**

In this exploratory and hypothesis-generating analysis, a small subset of patients with relapsing AAV showed suboptimal outcomes with rituximab. Residual disease activity at month 4, particularly in patients with PR3-ANCA positivity and ENT involvement, may represent a clinical subgroup associated with an increased frequency of earlier relapse, although this observation requires confirmation.

## 1 Introduction

ANCA-associated vasculitis (AAV), including granulomatosis with polyangiitis (GPA) and microscopic polyangiitis (MPA), comprises autoimmune disorders characterized by inflammation of small- to medium-sized blood vessels and may affect almost any organ system (1). Without treatment, mortality exceeds 90% within 2 years. The introduction of immunosuppressive therapy, initially glucocorticoids and cyclophosphamide and later rituximab, transformed AAV from a fatal disease into a chronic relapsing condition (2).

Management of AAV includes an induction phase (3–6 months) to suppress inflammation and prevent organ damage, followed by maintenance therapy to sustain remission, typically for at least 2 years (3). Remission rates with rituximab range from 64% to 83%, depending on trial response definitions, although a proportion of patients do not achieve complete remission or subsequently experience relapse (4–8).

Despite the overall efficacy of rituximab, less is known about patients who show suboptimal response to treatment, particularly in the setting of relapsing disease. In clinical practice and research, different scenarios—such as failure to achieve protocol-defined remission, incomplete remission at a fixed time point, and relapse during maintenance—are often considered together, although they may represent distinct biological and clinical states.

The aim of this study was to describe the frequency, characteristics, and outcomes of these scenarios of suboptimal response to rituximab in patients with relapsing AAV within the RITAZAREM trial (9, 10).

## 2 Methods

### 2.1 Study design

The RITAZAREM trial (NCT01697267) was an international, randomized, open-label superiority trial including 188 patients with relapsing GPA or MPA recruited from 29 centers in seven countries (2013–2016) (9, 10). At relapse, all patients received rituximab (375 mg/m^2^ weekly for 4 weeks) plus glucocorticoids. Those in remission at month 4 were randomized to maintenance with rituximab (1,000 mg every 4 months × 5) or azathioprine (2 mg/kg/day) until month 24. Follow-up lasted 36–48 months.

### 2.2 Study population and definitions

This was a *post hoc* descriptive analysis of the RITAZAREM trial. Three clinically defined scenarios were examined (Supplementary Figure 1):

- Population 1: Patients who did not achieve protocol-defined remission at month 4 and were not randomized (*n* = 6).
- Population 2: Patients with residual disease activity at month 4, defined as a BVAS/WG score of 1 with a prednisolone dose ≤ 10 mg/day (*n* = 10). Although these patients fulfilled the protocol-defined remission criteria of the trial (BVAS/WG ≤ 1), they were analyzed separately from those with complete remission (BVAS/WG = 0) to explore whether minimal residual disease activity was associated with distinct clinical outcomes. This subgroup should be regarded as an exploratory classification rather than a validated clinical phenotype.
- Population 3: Patients who relapsed during rituximab maintenance (*n* = 13), including three patients who had also been classified in Population 2.

These scenarios were analyzed descriptively and were not mutually exclusive. Population 1 was distinct, whereas Populations 2 and 3 partially overlapped because three patients with incomplete remission at month 4 subsequently relapsed during rituximab maintenance.

Protocol-defined remission was defined as a BVAS/WG score ≤ 1 together with prednisolone ≤ 10 mg/day at month 4, according to the RITAZAREM trial protocol. For the purposes of this *post hoc* analysis, patients meeting protocol-defined remission were further classified as having complete remission (BVAS/WG = 0) or incomplete remission (BVAS/WG = 1). The term “incomplete remission” is used solely for this exploratory subgroup analysis and does not represent an established disease category.

Baseline variables included age, sex, disease duration, prior organ involvement, previous cyclophosphamide or rituximab use, ANCA subtype, and disease manifestations.

ANCA positivity was determined using centrally assessed PR3-ANCA or MPO-ANCA results when available and otherwise using local trial laboratory results, as previously described for the RITAZAREM trial. A positive result was defined as an antibody index ≥ 1.0. Peripheral CD19+ B-cell measurements were performed at participating centers and recorded as absolute counts and/or percentages. For the present analysis, B-cell presence was defined as a CD19+ cell count ≥ 0.01 × 10^9^/L or ≥1% of lymphocytes. For Populations 1 and 2, ANCA and CD19+ B-cell status were assessed using samples obtained at the month 4 assessment. For Population 3, biomarker status was assessed using samples obtained at the time of relapse. Although semiquantitative ANCA measurements were available, only ANCA positivity was analyzed in the present study.

### 2.3 Statistical analysis

This was an exploratory descriptive analysis. Continuous variables are presented as mean (SD), and categorical variables as frequencies (%). Time to relapse from month 4 in patients with BVAS/WG = 1 versus BVAS/WG = 0 was explored using Kaplan–Meier curves and the log-rank test. Analyses were performed using SPSS v29.

## 3 Results

### 3.1 Population 1

Six of 188 patients (3%) did not achieve protocol-defined remission by month 4. Two patients showed worsening BVAS/WG scores, one had an insufficient response (BVAS/WG reduced from 6 to 2), and three relapsed after initial improvement. Mean BVAS/WG at month 4 was 3 (range 2–6), and three patients had at least one major item.

At month 4, ANCA remained detectable in four patients and CD19+ B cells were present in four (Supplementary Table 1).

Rescue treatment was individualized at the discretion of the treating investigator and was initiated at different time points before, around, or after the month-4 assessment, according to the persistence, worsening, or recurrence of disease activity. All six patients received increased or prolonged glucocorticoid therapy. Additional treatments included intravenous cyclophosphamide in two patients, plasma exchange in one, mycophenolate mofetil in one, and methotrexate in two. In one patient with worsening renal disease, plasma exchange and intravenous cyclophosphamide had already been initiated before month 4. One patient subsequently received rituximab outside the trial protocol. All six patients eventually achieved remission and were followed for a mean of 42.9 months (SD 3.1). During follow-up, five experienced at least one relapse, accounting for nine relapses (two major, seven minor). The mean time to first relapse was 6.8 months (SD 9.6).

### 3.2 Population 2

Ten of 188 patients (5.3%) met protocol-defined remission (BVAS/WG ≤ 1 and prednisolone ≤ 10 mg/day) but still had low-grade disease activity (BVAS/WG = 1) at month 4. All 10 patients had a history of PR3-ANCA positivity and experienced baseline ear/nose/throat (ENT) symptoms (Tables 1, 2). Among these patients, residual BVAS/WG items at month 4 were heterogeneous and included persistent hematuria (*n* = 3), persistent conductive hearing loss (*n* = 3), persistent orbital mass (*n* = 1), persistent headache (*n* = 1), persistent arthralgia (*n* = 1), and new/worsening pulmonary nodules (*n* = 1). Nine patients had persistent BVAS/WG items, whereas one patient had new/worsening disease activity. Baseline characteristics of patients randomized to rituximab who experienced a relapse during the maintenance phase were descriptively compared with those of patients who remained relapse-free (Supplementary Table 2).

**TABLE 1:** Baseline demographics of the RITAZAREM cohort and the different study populations.

Baseline demographics	Whole cohort (188 patients)	Population 1 (6 patients)	Population 2 (10 patients)	Population 3 (13 patients)
Age, years: mean (SD)	57.2 (14.9)	46.7 (11.1)	48.1 (13.8)	50.1 (14.9)
Male, number (%)	93 (49.7)	3 (50)	8 (80)	6 (46.2)
Race, number (%)				
White	167 (89.3)	3 (50)	9 (90)	11 (84.6)
Asian	13 (7.0)	2 (33.3)	1 (10)	1 (7.7)
Hispanic	3 (1.6)	0	0	0
Black	1 (0.5)	0	0	0
Other	3 (1.6)	1 (16.7)	0 (0)	1 (7.7)
Disease duration, months: mean (SD)	87.4 (81)	73.2 (124)	100.2 (100.4)	49.0 (34.5)
Prior cyclophosphamide (oral or/and IV), number of patients (%)	148 (79.1)	6 (100)	7 (70)	9 (69.2)
Prior treatment with rituximab, number of patients (%)	67 (35.8)	3 (50)	4 (40)	6 (46.1)
Glucocorticoid induction regimen (starting dose)				
1 mg/kg/day	54 (28.9)	0	4 (40)	5 (38.5)
0.5 mg/kg/day	133 (71.1)	6 (100)	6 (60)	8 (61.5)
ANCA type				
Anti-PR3	137 (73.3)	4 (66.7)	10 (100)	9 (69.3)
Anti-MPO	50 (26.7)	2 (33.3)	0 (0)	4 (30.7)
Relapse type on entry into trial				
Severe	120 (64.2)	4 (66.7)	4 (40)	8 (61.5)
Non-severe	67 (35.8)	2 (33.3)	6 (60)	5 (38.5)

SD, standard deviation; IV, intravenous; PR3, proteinase 3; MPO, myeloperoxidase.

**TABLE 2:** Clinical manifestations at the time of the trial inclusion of the RITAZAREM cohort and the different study populations.

Manifestations at baseline, number of patients (%)	Whole cohort (188 patients)	Population 1 (6 patients)	Population 2 (10 patients)	Population 3 (13 patients)
Constitutional	101 (54)	3 (50)	6 (60)	10 (76.9)
Joint	93 (49.7)	2 (33.3)	6 (60)	10 (76.9)
Skin	20 (10.7)	0	1 (10)	1 (7.7)
Mucous membranes/eyes	31 (16.6)	2 (33.3)	2 (20)	2 (15.4)
Ear/nose/throat	120 (64.2)	4 (66.7)	10 (100)	10 (76.9)
Cardiac	2 (1.1)	0	0	1 (7.7)
Gastrointestinal tract	0	0	0	0
Lung	69 (36.9)	3 (50)	6 (60)	2 (15.4)
Alveolar hemorrhage	19 (10.2)	0	0	0
Kidney	88 (47.1)	2 (33.3)	4 (40)	4 (30.9)
Nervous system	18 (9.1)	0	0	3 (23.1)

**FIGURE 1:**
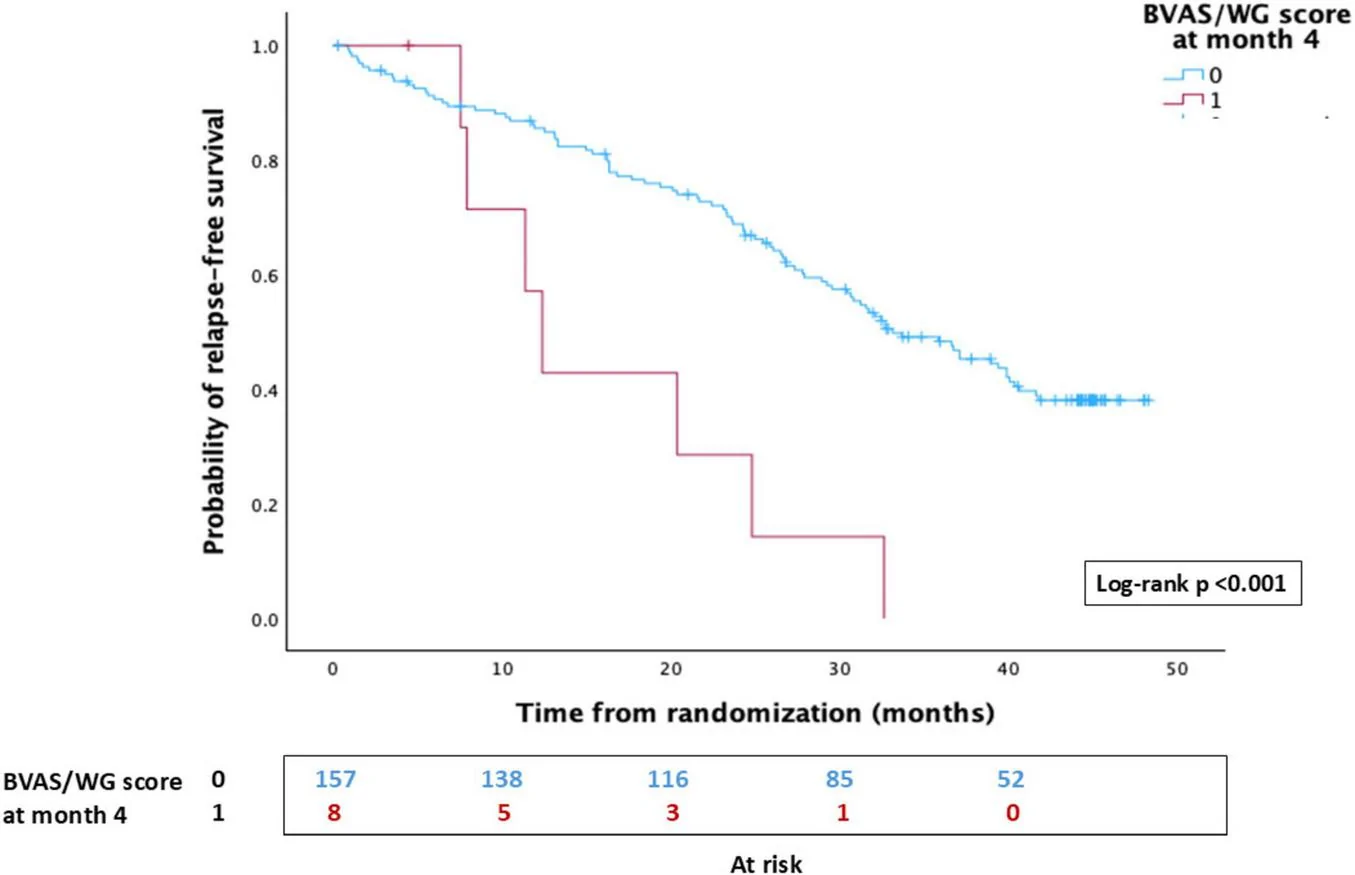
Probability of relapse-free survival in patients with a BVAS/WG score of 1 compared with patients with a score of 0 at month 4.

Of these 10 patients, eight were randomized into the maintenance phase (five to rituximab and three to azathioprine), while two were not randomized at physician discretion. Relapses occurred in 7/8 (87.5%), compared with 56.8% of patients who had achieved complete remission (BVAS/WG = 0) at month 4 (*p* = 0.06). Time to relapse was shorter (16.7 vs. 21.7 months), and Kaplan–Meier analysis showed shorter relapse-free survival (*p* < 0.001) in this subgroup (Figure 1), although interpretation is limited by the small sample size.

At month 4, ANCA remained detectable in four patients, whereas CD19+ B cells were absent in all (Supplementary Table 3).

### 3.3 Population 3

Among 85 patients receiving rituximab maintenance, 13 (15%) relapsed within 24 months. First relapses were minor in 10 and major in three, occurring at a mean of 9.8 months (SD 5.5). Minor relapses were treated with a protocolized short course of tapering prednisolone, starting at 20 mg/day. During follow-up after achieving remission, eight of these patients experienced a total of 10 subsequent relapses (six minor, four major).

At the time of relapse, ANCA positivity was detected in 2/13 patients and peripheral CD19+ B cells were present in 2/10 patients tested. One patient had both detectable ANCA and CD19+ B cells.

## 4 Discussion

This *post hoc* descriptive analysis of the RITAZAREM trial describes three clinically related but distinct scenarios of suboptimal outcome with rituximab in relapsing AAV. Although the use of rituximab to re-induce remission is highly effective for most patients with relapsing AAV, a small proportion of patients failed to achieve protocol defined remission (3%) or complete remission (8%) by month 4, while 15% of those receiving maintenance with rituximab relapsed within 24 months. Overall, these scenarios were uncommon, but they highlight that rituximab does not uniformly achieve sustained disease control in all patients. Given the small subgroup sizes and partial overlap between populations, findings should be interpreted as descriptive and hypothesis-generating.

Few studies in AAV have explored inefficacy or suboptimal response to rituximab. These studies have used varying definitions of remission, based on BVAS and prednisone dose, as well as different definitions of refractoriness, including disease progression, lack of improvement, or early relapse. The two pivotal clinical trials evaluating induction treatment with rituximab reported remission rates ranging from 64% at month 6 (defined as BVAS/WG of 0 and complete withdrawal of prednisone) (4) to 76% at month 12 (defined as BVAS/WG of 0 for at least 6 months while still on low-dose prednisone) (5). A *post hoc* analysis of the rituximab arm in the RAVE trial (11) showed that remission rates increased to 71% when patients receiving low-dose prednisone (mean 9.3 mg/day) were included, despite having a BVAS/WG of 0. Interestingly, that same analysis showed that 7% of patients (all PR3-ANCA positive) experienced worsening of AAV within the first few weeks (11). In the present analysis, six patients did not achieve protocol-defined remission by month 4, without clear distinguishing baseline characteristics, although the small sample size limits meaningful comparisons. In contrast, all patients with incomplete remission at month 4 had a history of PR3-ANCA positivity and baseline ENT involvement. However, because no multivariable analysis was performed and the incomplete remission subgroup was small, these findings should not be interpreted as evidence of independent predictors or causal relationships. Rather, they may reflect the broader PR3-positive GPA phenotype, which has consistently been associated with ENT involvement and an increased risk of relapse in previous studies (4, 7, 11–13), rather than a distinct rituximab-response phenotype. Larger prospective studies will be required to disentangle these potentially interrelated factors.

The clinical interpretation of residual disease activity (BVAS/WG = 1) at month 4 in the RITAZAREM trial requires caution. Residual disease activity at a fixed time point may reflect persistent disease activity, granulomatous manifestations less responsive to rituximab, incomplete suppression within the protocol-defined evaluation window, or simply a surrogate of disease phenotype, as suggested in previous studies examining remission definitions and relapse risk in AAV (4, 11–17). In particular, granulomatous manifestations, especially in ENT involvement, have been reported to be less responsive to rituximab compared with vasculitic features in some studies (4, 5, 12). In our cohort, most patients in the incomplete remission subgroup had persistent rather than new/worsening BVAS/WG items, including hematuria, conductive hearing loss, orbital mass, headache, and arthralgia, whereas only one patient had new/worsening pulmonary nodules. However, the available trial data do not allow us to determine whether these persistent findings reflected ongoing inflammatory activity or irreversible damage. This distinction is particularly challenging for ENT manifestations such as conductive hearing loss, although other persistent manifestations, including hematuria, orbital mass, or arthralgia, may also represent either active disease or residual damage depending on the clinical context. Consequently, some degree of misclassification cannot be excluded. Nevertheless, the high frequency of relapse observed in this subgroup suggests that patients with residual BVAS/WG activity at month 4 may represent a clinically relevant population requiring closer follow-up. These findings should be interpreted very cautiously, warranting confirmation in prospective studies specifically designed to characterize the nature and prognostic significance of minimal residual disease activity.

In the current analysis, after achieving remission, 13 (15%) patients receiving rituximab in the maintenance phase in the RITAZAREM trial experienced relapse. This is consistent with the 15.8% of patients who experienced relapse during the MAINRITSAN trial (14) and two recent retrospective studies (15, 16). Most relapses were initially minor, and a proportion of patients experienced further relapses after achieving remission with a short course of glucocorticoids. While this pattern may suggest a tendency toward recurrent disease activity in some patients, this analysis does not evaluate alternative treatment strategies or their impact on outcomes and, therefore, does not support specific management recommendations.

In the current analysis, biomarker findings in terms of persistent ANCA positivity and/or detectable CD19+ B cells should also be interpreted cautiously given the small sample size and the descriptive nature of the study. Although these biomarkers were present in some patients with suboptimal response, relapse also occurred in patients with negative ANCA and undetectable peripheral B cells, highlighting the heterogeneity of relapse mechanisms and the limited utility of these biomarkers in isolation. Previous studies have yielded inconsistent results regarding the utility of serial ANCA measurements and peripheral B-cell monitoring for predicting relapse. Observational studies have suggested that relapse often follows B-cell reconstitution and rising PR3-ANCA levels, supporting the potential role of biomarker-guided retreatment in selected patients (18). However, subsequent studies have shown that the predictive value of ANCA varies according to disease phenotype, being greater in patients with renal involvement than in those with predominantly non-renal disease (19). Furthermore, the MAINRITSAN2 trial demonstrated that an individually tailored rituximab maintenance strategy based on ANCA titres and B-cell repopulation was not superior to fixed-schedule retreatment (20). Taken together, these findings suggest that neither ANCA positivity nor peripheral B-cell repopulation alone reliably predicts relapse. Our findings are consistent with this concept and support the view that clinical assessment remains central when evaluating relapse risk, while ANCA and B-cell monitoring may provide complementary information in selected clinical contexts.

Strengths of this study include the use of prospectively collected data from a large, international, randomized trial with standardized definitions and follow-up (9, 10). However, several limitations should be considered. First, subgroup sizes were very small, limiting statistical power. Second, the analysis was *post hoc* and descriptive. Third, the analyzed scenarios are not independent, as some patients contributed to more than one group. Finally, rescue treatments were heterogeneous, were initiated at different points during follow-up, and were individualized at the discretion of the treating investigators rather than protocolized.

## Conclusion

Rituximab remains a cornerstone therapy and is highly effective in relapsing AAV. Nevertheless, a minority of patients experience suboptimal outcomes, including failure to achieve complete remission after induction or relapse despite rituximab maintenance therapy. In this small exploratory *post hoc* analysis of the RITAZAREM trial, patients with incomplete remission (BVAS/WG = 1) at month 4 had a higher observed frequency of relapse during follow-up. All patients in this subgroup had a history of PR3-ANCA positivity and baseline ENT involvement, although these findings may reflect the broader PR3-positive GPA phenotype rather than a distinct rituximab-response phenotype. These observations suggest that patients with minimal residual disease activity after rituximab induction may represent a clinically relevant subgroup warranting closer follow-up. However, given the small sample size and exploratory nature of the analysis, these findings should be considered hypothesis-generating and require confirmation in larger prospective studies.

## Data Availability

The datasets analyzed in this study are not publicly available because they contain data from the RITAZAREM clinical trial and are subject to ethical and data-sharing restrictions. Requests to access the datasets should be directed to the corresponding author. Data may be made available to qualified researchers upon reasonable request and with permission from the RITAZAREM investigators, in accordance with applicable institutional, ethical, and regulatory requirements.
