# Supplementary material for: Clinical scenarios of suboptimal response to rituximab in relapsing ANCA-associated vasculitis
Source: Front Med (Lausanne). 2026 Aug 31;13:1874867. doi: 10.3389/fmed.2026.1874867 (PMC13586283; doi:10.3389/fmed.2026.1874867)
Supplement: Supplementary file 1 [file Data_Sheet_1.docx]

**Supplemental figure 1**. Patient selection for the post hoc analysis from the RITAZAREM trial.

**Supplementary Table S1**. ANCA status and peripheral B cell counts in population 1 and the rest of the cohort at month 4.

| **Number of patients, (%)** | **Population 1**  **(n=6)** | **Rest of the cohort**  **(n= 172)** |
| --- | --- | --- |
| ANCA positivity | 4 (66.7%) | 84 (48.8) |
| Presence of CD19+ B cells | 4 (66.7%) | 16 (9.3) |
| ANCA positivity **or** presence of CD19+ B cells | 5 (83.3%) | 89 (51.7) |
| ANCA positivity **and** presence of CD19+ B cells | 3 (50%) | 10 (5.8) |

***Population 1*** *included patients who did not achieve protocol-defined remission protocol-defined remission after induction therapy and did not proceed to randomization. The “rest of the cohort” refers to randomized patients in the RITAZAREM trial with available ANCA and CD19+ B-cell data at month 4.*

*The presence of B cells (binary) was defined as either ≥ 0.01 cells x109/L or ≥ 1%.*

**Supplementary Table 2.** Baseline characteristics of patients randomized to rituximab according to relapse status during the maintenance phase.

|  | **Relapse**  (13 patients) | **No Relapse**  (72 patients) |
| --- | --- | --- |
| Age, years: mean (SD) | 50.1 (14.9) | 58.1 (115.3) |
| Male, number (%) | 6 (46.2) | 36 (50) |
| Race, number (%)  White  Asian  Hispanic  Black  Other | 11 (84.6)  1 (7.7)  0  0  1 (7.7) | 68 (94.4)  3 (4.2)  1  0  1 (1.4) |
| Disease duration, months: mean (SD) | 49.0 (34.5) | 101.2 (91.2) |
| Prior cyclophosphamide (oral or/and IV),  number of patients (%) | 9 (69.2) | 58 (80.6) |
| Prior treatment with rituximab,  number of patients (%) | 6 (46.1) | 27 (37.5) |
| Glucocorticoid induction regimen (starting dose)  1 mg/kg/day  0.5 mg/kg/day | 5 (38.5)  8 (61.5) | 19 (26.4)  57 (73.6) |
| ANCA type  Anti-PR3  Anti-MPO | 9 (69.3)  4 (30.7) | 53 (73.6)  19 (26.4) |
| Relapse type on entry into trial  Severe  Non-severe | 8 (61.5)  5 (38.5) | 44 (61.1)  28 (38.9) |
| Manifestations at baseline  number of patients (%)  Constitutional  Joint  Skin  Mucous Membranes/Eyes  Ear/nose/throat  Cardiac  Gastrointestinal tract  Lung  Alveolar hemorrhage  Kidney  Nervous system | 10 (76.9)  10 (76.9)  1 (7.7)  2 (15.4)  10 (76.9)  1 (7.7)  0  2 (15.4)  0  4 (30.9)  3 (23.1) | 31 (43.1)  28 (38.9)  7 (9.7)  12 (16.7)  48 (66.7)  1 (1.4)  0  25 (34.7)  6 (8.3)  35 (48.6)  6 (8.3) |

**Supplementary Table S3**. ANCA status and peripheral B cell counts in population 2 and the rest of the randomized cohort at month 4.

| **Number of patients, (%)** | **Population 2**  **(n=8)** | **Rest of the randomized cohort**  **(n= 162)** |
| --- | --- | --- |
| ANCA positivity | 4 (50%) | 79 (48.7) |
| Presence of CD19+ B cells | 0 | 16 (9.8) |
| ANCA positivity **or** presence of CD19+ B cells | 4 (50%) | 84 (51.8) |
| ANCA positivity **and** presence of CD19+ B cells | 0 | 10 (6.2) |

***Population 2*** *in the table includes data from the 8 randomized patients with BVAS/WG = 1 (2 patients in this population were not randomized at their physician discretion).*

*The presence of B cells (binary) was defined as either ≥ 0.01 cells x109/L or ≥ 1%.*
